# Generation and immune-characterization of single chain fragment variable (scFv) antibody recognize breast cancer cells line (MCF-7)

**DOI:** 10.1186/2051-1426-2-S1-P6

**Published:** 2014-02-24

**Authors:** Ilham Mahgoub, Ahmed K Bolad, Mohamed Mergani

**Affiliations:** 1 Microbiology and Unit of Immunology, Alneelain Medical Research Center, Faculty of Medicine, Khartoum, Sudan; 2 Biotechnology Engineering, International Islamic university Malaysia (IIUM), Kuala Lampour, Malaysia

## 

Phage display technologies were used to produce single-chain antibodies (scFv) against breast cancer cell line (MCF-7). The mouse B cell hybridoma line C3A8 was the starting material, which generates a monoclonal antibody against breast cancer cells (MCF-7). The best candidate scFv sequences, based on (ELISA) screening data were sub-cloned into HB2151 host strain. The scFv gene was expressed in E. coli cytoplasm for further analysis. The purified scFv protein was characterized using western blot, flow Cytometery, indirect ELISA and immunofluorescence tests. Bioinformatics tools were used to predict heavy (VH) and light (VL) chains models. In the results, the protein was showed specific binding toward MCF-7 cells line when a band of 68kD was appeared in Western blot test. Further, scFv clearly recognized the MCF-7 antigen epitopes in immuno-fluorescence test. Additionally, 99% of the cells numbers were bound to scFv protein as measured by flow cytometery analysis. The predicted structures of heavy and light chains were found to be acceptable and were used to predict sequential epitopes that docked against epidermal growth factor receptor on surface of MCF-7. Herein, the recombinant antibody technology is a rapid and effective approach to next generation of cancer diagnosis and immunotherapy.

